# Widespread variation in transcript abundance within and across developmental stages of *Trypanosoma brucei*

**DOI:** 10.1186/1471-2164-10-482

**Published:** 2009-10-19

**Authors:** Bryan C Jensen, Dhileep Sivam, Charles T Kifer, Peter J Myler, Marilyn Parsons

**Affiliations:** 1 Seattle Biomedical Research Institute, 307 Westlake Ave. North, Seattle, WA, 98109 USA; 2 Department of Medical Education and Biomedical Informatics, University of Washington, Seattle, WA, 98195 USA; 3 Department of Global Health, University of Washington, Seattle, WA, 98195 USA

## Abstract

**Background:**

*Trypanosoma brucei*, the causative agent of African sleeping sickness, undergoes a complex developmental cycle that takes place in mammalian and insect hosts and is accompanied by changes in metabolism and cellular morphology. While differences in mRNA expression have been described for many genes, genome-wide expression analyses have been largely lacking. Trypanosomatids represent a unique case in eukaryotes in that they transcribe protein-coding genes as large polycistronic units, and rarely regulate gene expression at the level of transcription initiation.

**Results:**

Here we present a comprehensive analysis of mRNA expression in several stages of parasite development. Utilizing microarrays that have multiple copies of multiple probes for each gene, we were able to demonstrate with a high degree of statistical confidence that approximately one-fourth of genes show differences in mRNA expression levels in the stages examined. These include complex patterns of gene expression within gene families, including the large family of variant surface glycoproteins (VSGs) and their relatives, where we have identified a number of constitutively expressed family members. Furthermore, we were able to assess the relative abundance of all transcripts in each stage, identifying the genes that are either weakly or highly expressed. Very few genes show no evidence of expression.

**Conclusion:**

Despite the lack of gene regulation at the level of transcription initiation, our results reveal extensive regulation of mRNA abundance associated with different life cycle and growth stages. In addition, analysis of variant surface glycoprotein gene expression reveals a more complex picture than previously thought. These data provide a valuable resource to the community of researchers studying this lethal agent.

## Background

*Trypanosoma brucei *subspecies are unicellular pathogens that infect humans, as well as domestic and wild animals. The infections of humans are fatal without treatment, and the treatments are suboptimal due to resistance, toxicity, and cost. Furthermore, no vaccine is available due to rampant antigenic variation in which hundreds of variant surface glycoproteins (VSGs) are sequentially displayed on the parasite surface [1]. Additionally, this group of organisms, along with their relatives *Trypanosoma congolense *and *Trypanosoma vivax*, have hampered the development of sub-Saharan Africa by their severe effects on cattle and draft animals.

In both the mammalian host and the insect vector (tsetse fly), *T. brucei *undergoes multiple developmental changes that are reflected by changes in morphology, surface proteins, cell division, and metabolism, although all stages are extracellular and motile, courtesy of a single flagellum. When first injected into the mammalian host by the bite of a tsetse fly, the stationary metacyclic phase parasites re-enter the cell cycle as rapidly dividing slender bloodstream forms (BF). This stage relies on glycolysis for the generation of ATP, and has a single tubular mitochondrion. After several days, short stumpy forms appear. These forms are arrested in G_0_/G_1 _and still express VSG, but show modest metabolic changes that appear to presage the next stage of parasite development [2-4]. When taken up by the fly, they readily transform into procyclic forms (PF) that multiply in the insect midgut. This stage of the parasite expresses a different surface coat, made up of a small family of proteins termed procyclins [5]. Glycolysis is down-regulated, amino acid metabolism is induced, and the mitochondrion enlarges and more elaborate cristae develop. After one to two weeks, the PFs journey to the salivary glands where they transform into epimastigotes and then into mammalian-infective metacyclic forms [6]. While *T. brucei *slender BFs and PFs are routinely cultured *in vitro*, epimastigotes and metacyclic forms are not readily available.

In contrast to most other organisms, trypanosomatids do not regulate gene expression at the transcriptional level, except for the major surface antigens of African trypanosomes such as *T. brucei *[7]. Almost all genes are transcribed as large polycistronic clusters, but adjacent genes may show widely differing levels of mRNA, and these levels may vary during the parasite life cycle. This developmental regulation of mRNA abundance is largely attained *via *changes in decay rate, predominantly attributed to sequences within the 3' untranslated region (UTR) [8]. Early studies of changes in gene expression between slender BF and PF suggested that only about 2% of probes showed significant changes in signal between stages [9,10]. However, these initial studies relied on arrays that utilized relatively large anonymous fragments of the genome as probes (~2.25 kb), and used a cutoff of 2.5-fold change between minimum and maximum signal. The elucidation of the complete genome of *T. brucei *strain 927 in 2005 [11] affords the opportunity to examine global changes in mRNA expression using a gene-specific approach. A recent study examined a subset of about 550 genes for their expression in BF as compared to PF parasites [12]. The genes were selected on the basis of their potential for regulation, based on analysis of the literature, and many were found to be stage-regulated. More recently, microarrays have been used to search for changes in gene expression following knockdown or knockout of genes encoding RNA binding proteins [13,14].

Until now, the microarray studies performed on *T. brucei *have been either sub-genomic or have utilized a single probe for each gene. In this study, we have used Nimblegen microarrays that employed eight probes per gene to assess gene expression in *T. brucei *927, examining the stages which are readily obtained in the laboratory. Those stages included *in vitro *cultured slender BF (cBF), as well as slender and stumpy BF harvested from infected rats. We also studied both log and stationary phase PFs (PF-log, PF-stat). Our results revealed that mRNA expression in cBF was little different from that seen in the slender BFs harvested from rats. A modest number (~100) of changes were seen in stumpy BF. As expected, many changes were observed when cBF and PF-log parasites were compared, with several hundred genes showing at least a 2-fold change in mRNA abundance. Surprisingly, comparison of PF-log and PF-stat parasites showed an even larger number of changes. The arrays not only allowed us to compare differences between stages, but also to examine expression levels of various genes within a sample. About half of genes ranked in the top 10% of signals in cBF were also expressed to high levels in all other stages. A significant fraction of these highly expressed genes encoded hypothetical proteins. Very few genes showed no evidence of expression.

## Results and Discussion

This study reports our findings on changes in gene expression between those stages of *T. brucei *that can be readily studied in the laboratory. During natural infections in the vertebrate host, *T. brucei *progresses from the actively dividing slender BF to the non-dividing stumpy BF. This form is primed for differentiation to procyclic forms in the insect host. We examined the expression level of nearly all predicted protein-coding genes of strain 927 *T. brucei*, as well as many RNA genes, in five different parasite populations. Most of the analyses reported here are restricted to nuclearly encoded transcripts, although some findings for mitochondrially derived transcripts are noted. Three biological replicates were used for each test condition. Three of the five sets were derived from *in vitro *culture under highly standardized conditions: log-phase cultured bloodstream forms (cBF), log-phase cultured procyclic forms (PF-log), and stationary-phase procyclic forms (PF-stat) (see Additional file 1). The remaining two samples, slender BF and stumpy BF, were derived from infected rats and were likely to show more inter-sample variability, since they were grown for varying times in rats, and the animals became progressively less healthy after irradiation and infection. Since the stumpy BF populations were the last to be harvested and each contained a variable proportion of intermediately differentiated forms, these populations were the most biologically variable. They ranged from 76% to 93% morphologically stumpy and 68% to 88% of cells expressed the PF marker EP procyclin upon appropriate stimulation (see Table 1). The slender BF populations showed less than 5% intermediate or stumpy forms and less than 1% expressed procyclin after stimulation.

**Table 1:** Characterization of bloodstream form *T. brucei *from animals

**Cell type**	**Parasitemia**	**Cell morphology**			**procyclin staining**	
	**(cells/ml)**	**Slender**	**Intermediate**	**Stumpy**	**positive**	**negative**
Slender	5.6 × 10^7^	97%	3%	0%	0%	100%
	1.2 × 10^8^	97.6%	2.4%	0%	0%	100%
	9.4 × 10^7^	94.5%	5.5%	0%	1.0%	99.0%

Stumpy	5.2 × 10^8^	0%	20.7%	79.3%	88.2%	11.8%
	6.1 × 10^8^	0.6%	6.4%	93%	89%	11%
	6.9 × 10^8^	5.1%	18.5%	76.4%	67.5%	32.5%

The Nimblegen arrays that were hybridized with cDNA prepared from each parasite population contained multiple probes per gene (in most cases, eight), and three copies of these probe-sets per chip. After normalization to allow for cross-chip comparisons, we calculated a single value for each set of triplicate probes using Tukey's biweight formula. We then obtained a single gene level value using the Tukey biweight of the signals for the probes corresponding to each gene (Additional file 2). In most cases, this value represented the "average" of 72 data points collected for every protein-coding gene (45 for RNA genes) in each of the five growth conditions. Thus, as detailed in Methods, we were able to utilize robust statistical analyses that increase our confidence in the findings of the comparisons reported below.

### Gene expression levels

**Figure 1:**
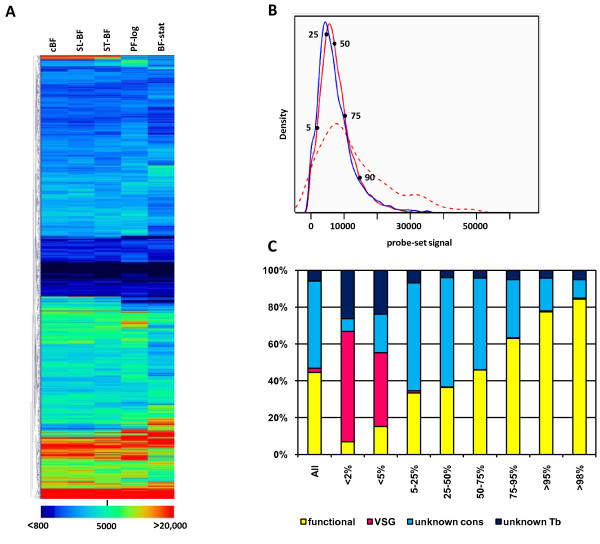
**Distribution of different expression levels for all *T. brucei *nuclear genes**. **A**. Heatmap showing the signals obtained for cBF, slender BF (SL-BF), stumpy BF (ST-BF), PF-log), and PF-stat. **B**. Density plot of gene level intensities for nuclear protein-coding genes. Blue line, PF-stat; red line, cBF; dashed red line, cBF signals from the multicopy probe-sets. The points mark the signals in cBF corresponding to the percentile rank indicated. **C**. Distribution of protein-coding gene categories varies according to expression level. The maximal expression level (Tukey mean) for each gene was ranked. Genes encoding proteins with an ascribed function or location are included in the functional category, the remaining genes were divided into those specific to *T. brucei *(unknown Tb) and those conserved in at least one other species (unknown cons).

The normalized expression values from the 8110 probe-sets corresponding to nuclear genes were hierarchically clustered using the TMeV software package and are shown graphically as a heat map in Figure 1A. A small number of genes (those colored black or dark blue in the middle of the figure) showed no or very low expression under any condition, and a similar number (colored red) showed high expression levels in some (top) or all (bottom) conditions. However, the large majority of the genes showed low (blue) or moderate (green or yellow) expression levels. Each biological sample set showed a similar distribution of signal intensities obtained for the 8004 probe-sets corresponding to nuclear protein-coding genes, with the curves obtained from cBF and PF-stat (the most divergent samples) shown Figure 1B. This figure also depicts the signals obtained for the 345 probe-sets that map to multicopy CDSs. This curve was skewed dramatically to the right, as compared to the total sample of probe-sets, confirming gene amplification as a clear strategy for increasing gene expression in *T. brucei*. Indeed, 20 of the 50 probe-sets detecting the highest signals map to two or more genes (see below and Table 2). However, the signals did not follow in rank order of number of genes detected by the probe-sets.

**Table 2:** The 50 most highly expressed protein-coding genes

**Rank**	**name**	**Gene #^a^**	**Description**	**Max**	**Max_stage^b^**
1	Tb927.1.4620	1	hypothetical conserved	64153	cBF
2	Tb927.8.7410	1	calreticulin	56648	slender BF
3	Tb927.1.2380	4	alpha tubulin	55277	slender BF
4	Tb10.6k15.0020	1	EP1 procyclin	53583	PF-stat
5	Tb927.6.510	1	GPEET2 procyclin precursor	52410	PF-log*
6	Tb927.4.5010	1	calreticulin	51616	cBF*
7	Tb927.1.2350	4	beta tubulin	50396	PF-log
8	Tb10.70.1370	1	fructose-bisphosphate aldolase glycosomal	50161	slender BF*
9	Tb10.406.0390	11	histone H2B	49321	slender BF
10	Tb927.5.2260	1	hypothetical conserved	49312	PF-log*
11	Tb927.1.2560	7	hypothetical	49217	PF-log
12	Tb11.02.4690	1	Hypothetical	48922	cBF
13	Tb10.6k15.2040	1	glucose transporter 1B	48339	slender BF*
14	Tb11.1190	1	hypothetical	48183	cBF
15	Tb927.1.4600	5	F-box motif protein, CFB1A-1E	47725	cBF*
16	Tb927.8.4710	3	amino acid transporter	47553	slender BF
17	Tb927.8.4700	2	amino acid transporter	47452	stumpy BF
18	Tb927.1.2530	7	histone H3	47213	slender BF
19	Tb927.4.4730	1	amino acid transporter	46274	PF-stat*
20	Tb11.01.3110	1	heat shock protein 70	45565	PF-log
21	Tb11.01.7800	1	nucleoside diphosphate kinase	45393	PF-log
22	Tb927.5.1810	1	p67 lysosomal membrane glycoprotein	44412	stumpy BF
23	Tb927.7.6040	2	Hypothetical	44288	slender BF
24	Tb927.8.5260	1	60S ribosomal protein L39	44187	PF-stat
25	Tb10.406.0360	2	histone H2B	44099	slender BF*
26	Tb11.01.0355	1	ribosomal protein S26	43926	PF-stat
27	Tb927.7.2930	13	histone H2A	43798	slender BF
28	Tb09.160.5400	1	ESAG9	43669	stumpy BF*
29	Tb10.70.5650	3	elongation factor 1-alpha TEF1	43424	cBF
30	Tb09.244.2740	2	60S ribosomal protein L5	42964	PF-log
31	Tb927.8.8300	1	amino acid transporter	42640	PF-stat*
32	Tb10.70.3370	2	40S ribosomal protein S3a	42603	stumpy BF
33	Tb10.26.1080	1	heat shock protein 83	42429	PF-log
34	Tb927.1.580	1	phosphate-repressible phosphate permease	42138	PF-stat
35	Tb927.6.970	9	cysteine peptidase precursor	41870	slender BF
36	Tb927.5.810	1	hypothetical conserved, zinc finger protein	41840	stumpy BF
37	Tb10.6k15.0030	1	EP2 procyclin	41304	PF-stat*
38	Tb10.6k15.2020	1	glucose transporter 2A	40774	PF-stat
39	Tb10.v4.0052	1	microtubule-associated protein 2	40337	cBF
40	Tb09.211.0340	2	60S ribosomal protein L10	40302	PF-stat
41	Tb10.70.2650	2	elongation factor 2	39867	PF-log*
42	Tb927.4.1860	1	ribosomal protein S19	39840	PF-stat
43	Tb927.8.5460	3	flagellar calcium-binding protein 44 kDa	39840	cBF
44	Tb927.4.1800	2	ribosomal protein L3 mitochondrial	39831	PF-stat
45	Tb927.8.6180	1	60S ribosomal protein L26	39793	stumpy BF
46	Tb927.8.6450	1	inhibitor of cysteine peptidase chagasin	39785	stumpy BF*
47	Tb11.01.3180	2	guanine nucleotide-binding protein beta subunit-like	39559	PF-log
48	Tb927.5.1710	1	ribonucleoprotein p18, complex V	39457	PF-log
49	Tb10.406.0340	1	histone H2B	39390	cBF
50	Tb927.1.2310	1	Hypothetical	39078	PF-stat*

^a^number of genes detected by probe set

^b^Asterisk indicates gene is regulated at least two-fold between highest and lowest sample sets.

For the total probe-sets, there is a large peak centered at ~4600 for the PF-stat and ~5800 for cBF. In both cases, the peak moves sharply down towards lower values, with a small shoulder at ~200. When the same arrays were probed with RNA derived from a different strain, this shoulder was more pronounced, and a corresponding increase in probe-sets with signal intensities less than 200 was observed (unpublished data). Since many of these probe-sets mapped to *VSG*s, most of which are not conserved between strains, a signal level of ~200 can be taken as a generous estimate of background for non-transcribed regions. However, almost all regions of the *T. brucei *genome are thought to be constitutively transcribed, and hence even those genes whose mRNAs are unstable would likely show a signal higher than this background. The number of probe-sets that failed to show a signal level of less than four times the "non-transcribed background" signal (*i.e*., 800) in least one of the five different biological conditions was small (164 protein-coding genes). Even then, *VSG *genes, which are subject to clonal variations in expression, accounted for all but 65 of this set. The majority (55) of the remaining genes have unknown function, with most of these (44) found only in *T. brucei*, raising the possibility that they do not represent authentic genes. In addition, almost all of these low-expressing genes are located in sub-telomeric clusters of *VSG*s or expression site associated genes (*ESAG*s) or are immediately adjacent to convergent strand-switch regions where transcription terminates [15,16]. Even when considering only cBF parasites, only 133 predicted non-*VSG *protein-coding genes had a signal <800, and of these only 22 had annotated function. Notably, most of these genes were expressed to higher levels in at least one other stage, and those that were low in all stages were located in a sub-telomeric or convergent strand-switch region context.

The protein-coding genes were ranked according to their maximal expression level in any stage, and broadly categorized according to their annotated function (Figure 1C). As suggested above, *VSG *genes and *T. brucei*-specific genes of unknown function accounted for most of the genes showing the lowest expression (86% of the bottom 2% and 64% of the bottom 5%). Conversely, genes which are more highly expressed tend to have some type of functional annotation; indeed many of these have been studied experimentally.

**Figure 2:**
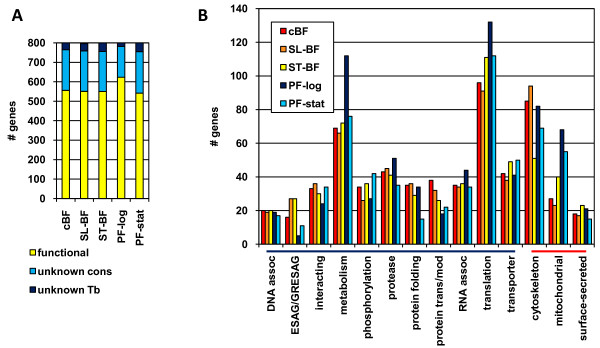
**Distribution of functional gene category in the top 10^th ^percentile of expression values in each biological condition**. **A**. Distribution of the functional gene categories as described in Figure 1C. **B**. Further breakdown of functional category. Only those categories with at least ten genes at one condition are shown. The functional (blue bar) and location (red bar) categories are indicated below the X-axis.

We identified the nuclear CDSs with the top 10% of signals for each biological condition. The genes were individually examined and placed into categories based on their annotation and/or their proteomic detection in specific sub-cellular fractions. Figure 2A shows the distribution of these genes into broad categories as in Figure 1C, while Figure 2B shows the distribution of genes with ascribed function (yellow in Figure 2A) according to various categories of biological function or location for the each of the five different biological conditions. As indicated in Figure 1C, above, genes with unknown function were under-represented in the highly expressed category, as compared to the whole genome, and this was most striking in the PF-log cells (Figure 2A). Overall, the categories with the largest numbers of highly expressed genes were translation, metabolism, or cytoskeleton, with the majority (99 out of 144) of the genes in the translation category encoding cytoplasmic ribosomal proteins. Genes involved in mitochondrial function were highly represented in the top expressors in the PF stages, but more modestly represented in BF stages. As expected, significantly higher proportions of *ESAG*s or genes related to them *(GRESAG*s) were expressed at high levels in all BF stages than in the PF stages. In general, relatively similar proportion of genes were found in each category in all biological stages, although the number of genes involved in metabolism was increased in PF-log and genes encoding cytoskeleton proteins decreased in stumpy BF. However, this does not mean that the same genes are expressed to high levels in all stages. For example, 13% of mRNAs highly expressed in cBF were more than 2-fold up-regulated as compared to PF and similarly 12% of mRNAs highly expressed in PF were more than 2-fold up-regulated as compared to cBF (see below).

Among the 800 most highly expressed nuclear CDSs in cBF, 307 were highly expressed in every condition examined. Surprisingly, 111 of these had unknown function. Thus, there is a large set of highly expressed *T. brucei*-specific (20) or conserved (91) genes that have not been ascribed a function; including nine of the 50 most highly expressed genes in cBF (see Table 2). Some of the "hypothetical" proteins encoded by these nine genes have been shown to exist by proteomic analyses (Tb11.01.2800, Tb10.6k15.1500) or other studies (Tb927.1.4600 [13]). Conversely, two other "hypothetical genes" lie in intergenic regions between coding regions that are highly expressed, raising the possibility that these are not separate genes, but rather simply sequences within 3' UTRs of the neighboring genes. The first, as represented by Tb927.1.2540, is one of a set of seven almost identical putative genes (six of which are annotated as "hypothetical protein, unlikely") that are interspersed between the histone H3 genes. The second, as represented by Tb927.1.4590, corresponds to a set of genes that are interspersed between the highly expressed *CFB1 *genes. Tb11.1190 encodes a putative protein which is composed of 49 repeats of 68 amino acids and is represented on the microarray by a single probe corresponding to a unique sequence at the C-terminus. Tb11.02.4690 specifies a 22 kDa protein with a signal sequence and three transmembrane domains. It is expressed to a much higher level than the flanking genes, indicating it is a distinct mRNA. The last of these nine genes, Tb927.4.1000, encodes a 25 kDa protein that is also expressed much more highly than the adjacent genes.

### Differential gene expression

**Figure 3:**
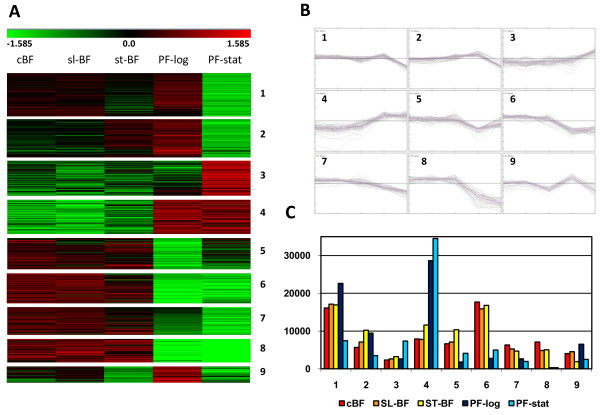
**Cluster analysis of highly-regulated mRNAs**. The 534 genes showing two-fold deviation from the mean expression value in at least one of the 5 samples using multi-class SAM analysis were analyzed. *ESAG*s, *GRESAG*s, *VSG*s, and *VR*s were excluded from the analysis. **A**. Heatmap. The genes were subjected to K-median clustering of the log_2 _ratios into 9 clusters, as numbered. **B**. Signals corresponding to individual probe-sets within each cluster. For each probe-set, the signal for each biological condition was compared to the mean of those signals across conditions (log_2 _ratio). The biological conditions from left to right are: cBF, slender BF, stumpy BF, PF-log and PF-stat. **C**. Tukey mean expression values across biological conditions for a single gene from each cluster, as numbered. The individual genes are: 1, Tb11.42.0003, t protein complex β subunit; 2, Tb10.61.1990, ribosome biogenesis protein; 3, Tb927.3.3990, RNA editing protein B6; 4, Tb09.160.1820, cytochrome oxidase subunit V; 5, Tb11.02.5610, GP63-1 surface protease; 6, Tb927.5.390, ISG75 invariant surface glycoprotein; 7, Tb10.70.3560, predicted RING finger protein; 8, Tb927.2.6240, adenosine transporter 2; 9, Tb927.7.6370, conserved hypothetical protein.

Comparison of the Tukey mean maximum and minimum signal levels for all probe-sets corresponding to nuclear genes revealed 122 genes that showed a greater than 10-fold change between two or more of the five biological conditions tested. Of these, 30 were *VSG*, *VSG*-related (*VR*) genes, or *ESAG/GRESAG*s, many of which are associated with antigenic variation. A total of 446 genes (including 161 *VSG*s and *ESAG*s) showed more than 4-fold variation, while at the 2-fold level, 2105 genes (including 233 *VSG*s and *ESAG*s) showed a statistically supported difference in expression. Thus, over one-fourth of all of the genes assessed on the microarray were differentially expressed (*i.e*. q-value of <5% in multi-class significance analysis of microarrays (SAM)) between at least two conditions. This dataset was further reduced to those showing a >2-fold deviation from the mean of all five conditions in at least one sample, and by excluding all genes encoding VSG/VRs and *ESAG/GRESAG*s (which were analyzed separately, see below). These 534 probe-sets were K-median clustered using settings indicated in the Methods to yield nine clusters which contained between 31 and 80 genes (Figure 3 and Additional file 3). Each of these clusters represents a distinct pattern of gene expression, although some are similar. Overall, these highly regulated genes are enriched in those involved in metabolism, proteolysis, translation and *T. brucei*-specific unknown functions, but under-represented in those genes that are conserved but have unknown function. However, individual clusters show differential enrichment in particular functional gene categories.

Figure 3A shows the heatmap depicting gene clustering, while Figure 3B shows overlaid expression patterns graphically for every gene in each cluster. Figure 3C depicts specific examples that illustrate the expression patterns characteristic of the gene clusters. Genes in clusters 5, 6, 7 and 8 all have higher mRNA levels in BF than in PF, although the clusters each show subtle differences in expression pattern. For example, cluster 8 contains genes that encode mRNAs substantially down-regulated in PF-log and PF-stat, whereas in cluster 6 the down-regulation in PF is more modest and some genes begin to decrease expression in stumpy BF. Cluster 8 is enriched in genes involved in metabolism and adenosine transport and also includes several genes encoding 64-65 kDa invariant surface glycoproteins (ISGs) and procyclin-associated genes (*PAG*s). Cluster 6 contains genes encoding three 75 kDa ISGs, several proteases, and a substantial number of *T. brucei*-specific proteins of unknown function. Cluster 7 contains genes that are somewhat down-regulated in PF-log (relative to BF), but are expressed at even lower levels in PF-stat. Genes encoding proteins involved in interaction, metabolism, protein folding and protein transport or modification, are overrepresented in this cluster. Conversely, cluster 5 contains 60 genes that are down-regulated to a greater extent in PF-log than in PF-stat. This cluster is dominated by genes encoding proteases and *T. brucei*-specific proteins with unknown function. The former category includes several paralogues encoding homologues of the *Leishmania *gp63 surface protease.

Clusters 2, 3, and 4 show different patterns of up-regulation with respect to the two PF biological conditions. Cluster 2 contains genes that are up-regulated in PF-log, but not in PF-stat. For some genes this change in expression begins in stumpy BFs. The genes in this cluster are over-represented for those encoding proteins involved in interaction, metabolism, RNA processing, transcription, translation and cytoskeleton function. Their reduced expression in PF-stat is consistent with cessation of growth functions upon entry into stationary phase. Indeed we observed a significant accumulation of rRNA precursors in the PF-stat samples upon analysis on the Agilent BioAnalyzer (not shown). Conversely, cluster 3 contains genes that are up-regulated only in PF-stat and mostly have unknown function, including eight that are *T. brucei*-specific. It is possible that some of these gene products are involved in preparation for differentiation into epimastigotes, the next stage in the parasite life cycle. Finally, cluster 4 contains genes with higher expression levels in both PF-log and PF-stat and is enriched in genes encoding proteins involved in metabolism, proteolysis, or with unknown function but located on the cell surface or mitochondrion. As discussed in more detail below, this is consistent with the switch to mitochondrial pathways for energy generation in procyclics.

Cluster 1 contains the largest number of genes, with 82 members. The expression of these genes is lower in PF-stat (and stumpy BF in some cases), but the genes are expressed at higher levels in cBF and slender BF than seen in cluster 2. These genes are over-represented in those involved in metabolism, DNA replication/repair, protein folding, proteolysis and translation, consistent with their down-regulation in stationary-phase cells. Cluster 9, with 31 members, is the smallest cluster. The pattern of gene regulation is similar to cluster 1, but with some up-regulation in PF-log. Like cluster 1, this cluster is enriched in genes involved in DNA replication/repair.

The existence of these varied expression patterns implies a complex set of regulatory mechanisms operating at the RNA level to control the abundance of transcripts encoded by nuclear genes. The specific proteins involved in these processes are only beginning to be examined (see for example refs. [13,14,17]).

In contrast to the analyses above that examined the transcripts showing the most variation in abundance, we also looked at the transcripts that showed the least variation. Genes such as these would provide excellent controls for studies of developmental changes in gene expression. We identified 830 genes with a maximum variation in expression between the five stages of <25% (see Additional file 2). As expected, genes encoding proteins involved in known stage-regulated processes such as glycolysis and electron transport are significantly under-represented. However, the group is slightly enriched for genes of unknown function. Genes encoding proteins involved in lipid or fatty acid metabolism are also over-represented, comprising 28% of the metabolic enzymes that showed little variation as opposed to 11% of all metabolic enzymes. Similarly, genes encoding proteins of the ubiquitin pathway represent 40% of all protease-related genes, but 85% of the subset of protease-related genes that showed little variation. Genes involved in histone acetylation or chromatin structure, such as Tb927.7.1690 and Tb927.4.2520 (which encode transcriptional silencer Sir2) also tended to maintain similar mRNA levels between life cycle stages.

### Comparison of cBF and log phase PF forms

In order to identify differences in gene expression between specific conditions, we conducted pair-wise comparisons of specific datasets (including cBF *versus *slender BF, slender BF *versus *stumpy BF, cBF *versus *PF-log, and PF-log *versus *PF-stat) using SAM, setting the q-value to <5% and the fold-change to >2 (see Methods). Because *VSG *expression is both clonal and highly variable, *VSG*s are excluded from the gene tallies below, unless otherwise noted.

**Figure 4:**
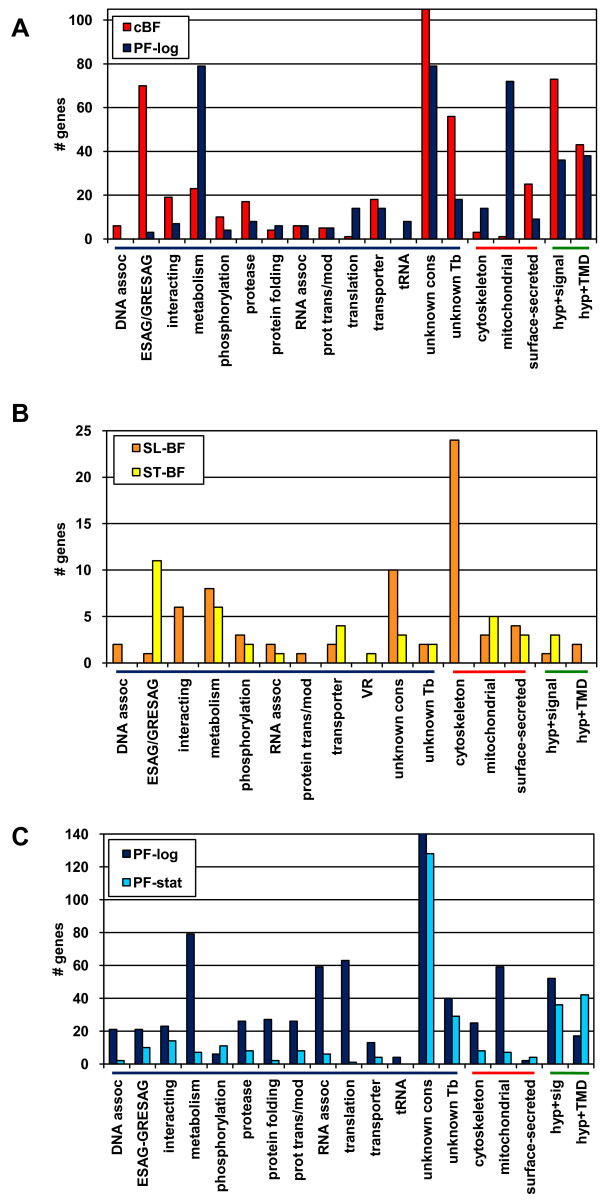
**Pairwise comparison of gene expression in different biological conditions**. Nuclear genes with >2-fold change in expression between the two conditions indicated (by either Tukey medians or SAM-calculated mean) were categorized into different functional categories or locations. Categories with very few representatives in either stage are not shown. Genes annotated as hypothetical or hypothetical conserved were further categorized as encoding proteins having a signal sequence (hyp+sig) or having at least one internal potential membrane domain (hyp +TMD).

Comparing the signals between cBF and PF-log, 691 genes were found to be differentially expressed. When the stringency of the SAM analysis was reduced to a 1.7-fold change, 963 genes were detected. A further reduction to 1.5-fold identified 1508 genes--approximately 19% of the genome. Thus, a relatively large fraction of the genome encodes mRNAs that differ in abundance between these two stages. Figure 4A shows a comparison of the functional categories of the genes showing >2-fold regulation; these are individually listed (along with their fold-changes in mRNA expression and q-values) in Additional file 4. Table 3 itemizes those genes upregulated in cBF that have predicted functions (excluding *VSG*s and *ESAG*S, which are discussed below).

**Table 3:** Genes with functional annotation that are up-regulated in cBF as compared to PF-log^a^

**Description**	**SysID (fold change^b^, signal in cBF)**
**Nucleic acid**	
cold shock DNA binding domain protein	Tb927.4.4520 (3.8, 11127), Tb927.8.7820(3.1, 30733)
DNA binding motif protein	Tb927.8.8270 (2.1, 5130)
methylated DNA binding motif protein	Tb09.160.1490 (2.1, 6911)
SNF2 DNA repair protein	Tb927.7.4650 (**3.6**, 9704)
DNA topoisomerase II	Tb11.01.3390 (2.2, 20052)
heterogeneous nuclear ribonucleoprotein H/F	Tb927.2.3880 (2.5, 19993)
RNA-binding protein	Tb927.6.3480 (4.6, 15068), Tb927.7.3730 (2.4, 19060), Tb927.8.2780 (**4.1**, 22483, Tb10.389.1640 (2.9, 4885), Tb11.01.3940 (2.9, 6039)
**Interaction Motifs**	
hypothetical conserved, ankyrin repeat	Tb11.01.6010 (**2.1**, 9639), Tb927.7.1420 (**2.2**, 13373)
hypothetical conserved, FHA and BRCT domains	Tb927.4.500 (**2.6**, 10222)
hypothetical conserved, PX domain	Tb927.7.4500 (2.3, 19542)
hypothetical conserved, RING finger	Tb10.70.3560 (**2.4**, 6310)
hypothetical conserved, zinc finger	Tb10.389.0740 (**7.6**, 16571), Tb10.70.2020 (**4.7**, 8391), Tb11.01.0220 (**2.7**, 20691), Tb10.70.1850 (2.7, 3725), Tb09.211.1720 (2.5, 18066), Tb927.3.5390 (2.3, 3358), Tb11.01.0090 (2.1, 19108), Tb11.02.2470 (**2.0**, 15163), Tb11.01.8270 (2.3,18420)
leucine-rich repeat protein	Tb11.02.1564 (**5.1**, 5086), Tb927.3.1490 (**4.8**, 3199), Tb927.3.580 (**3.2**, 7044), Tb11.02.1580 (**2.2**, 15932)
zinc-binding protein (Yippee)	Tb927.6.4810 (1.9, 3902)
**Metabolism**	
acidic phosphatase	Tb927.5.610 (3.9, 21844)
acidocalcisomal pyrophosphatase VSP1	Tb11.02.4930 (2, 21100)
alternative oxidase	Tb10.6k15.3640 (**5.2**, 25749)
arginine kinase	Tb09.160.4570+1 (2.1, 8792)
aspartate aminotransferase	Tb10.70.3710 (2.2,10132)
ATP-dependent phosphofructokinase	Tb927.3.3270 (**2.7**, 35922)
Diacylglycerol kinase catalytic domain	Tb927.8.5140 (**2.2**,16973)
sphingolipid delta 4 desaturase	Tb927.6.3000 (2.3,10540)
fructose-1,6-bisphosphatase	Tb09.211.0540 (**2.3**,13478)
glutathionylspermidine synthetase	Tb11.12.0016 (2.1, 20983)
guanine deaminase	Tb05.5K5.200+1 (2, 7271)
haloacid dehalogenase-like hydrolase	Tb11.01.0120 (**2.3**, 8482)
hexokinase 1	Tb10.70.5820* (**13.4**, 27920)
hypothetical conserved, serine-esterase like motif	Tb11.01.3580 (2.2, 10321)
iron/ascorbate oxidoreductase family protein	Tb927.2.6180 (**13.2**, 6052), Tb927.2.6230 (**6.9**, 13271), Tb927.2.6310 (**3.3**, 8181)
lipase domain protein	Tb927.3.3870 (**3.1**, 13228)
nucleoside phosphorylase	Tb927.8.4430 (3.2, 23275)
phosphoglycerate kinase, glycosomal	Tb927.1.700* (**10.1**, 32849)
pyruvate kinase 1	Tb10.61.2680* (**3.5**, 35164)
sphingomyelin synthase family	Tb09.211.1020 (3.1, 9771), Tb09.211.1010 (2.3,)12554
**Protein or lipid phosphorylation**	
casein kinase I, CK1	Tb10.70.5340 (**2.6**,12672)
cdc2-related protein kinase	Tb11.47.0031 (**3.1**, 15760)
cyclin 3, mitotic cyclin	Tb927.6.1460 (2.4, 11043)
dual specificity phosphatase	Tb11.02.1640 (2.3, 5100)
protein kinase	Tb927.5.3320 (2.4, 6629)
serine/threonine-protein kinase, NEK family	Tb927.2.2120 (2.2, 11101), Tb927.4.5310 (2.6, 13985), Tb927.8.7110 (2, 16132)
TFIIF-stimulated CTD phosphatase	Tb927.3.3380 (**3**, 8247), Tb10.61.2520 (2.5, 16649)
**Protease-related**	
calpain cysteine peptidase	Tb927.8.8330 (**3.3**, 19000)
cysteine peptidase C	Tb927.6.560 (**2**, 17546)
Gp63 major surface protease homolog	Tb11.02.5310 (**2.3**, 16249), Tb10.70.5290* (**5.2**,14867), Tb11.02.5630 (**4.2**, 10649), Tb11.02.5610 (**3.7**, 6645), Tb11.02.5640 (**3.3**, 12431), Tb11.0370 (**3.6**, 4695), Tb11.0380 (2.6, 10329), Tb11.0360 (**3.4**, 9307)
hypothetical conserved, OTU protease domain	Tb927.8.5050 (2.2, 13601)
hypothetical conserved, UIM domain	Tb10.70.1130 (**2.2**, 20751)
inhibitor of cysteine peptidase chagasin family	Tb927.8.6450 (2, 29643)
metacaspase	Tb11.02.0730 (2.4, 11810), Tb927.6.930 (**3**, 1906), Tb10.70.5250 (**15.1**, 15483)
serine carboxypeptidase (CBP1)	Tb10.70.7090 (**3.9**, 16383)
serine peptidase	Tb927.3.4230 (**2.7**, 11496)
signal peptidase type I	Tb927.5.3220 (2.2, 13695)
**Protein folding, modification and transport**	
acetyltransferase	Tb927.1.4490 (2.7, 4473)
ADP-ribosylation factor	Tb11.01.6060 (2.2, 4625)
chaperone protein DNAJ	Tb927.4.3980 (**3.4**, 10760), Tb927.6.3120 (2.5, 17514)
dynamin vacuolar sortin protein 1	Tb927.3.4720 (2.3, 13449)
GPI inositol deacylase precursor	Tb10.70.2420 (**2.6**, 21099)
heat shock protein HSP70-like protein	Tb09.160.3090 (**2.4**, 26203)
HSR1-related GTP binding protein	Tb927.4.2380 (2.0, 16054)
hypothetical conserved, TRAP alpha motif	Tb927.7.2190 (2.1, 3176)
oligosaccharyl transferase subunit	Tb927.5.890 (**2.9**, 10460)
protein disulfide isomerase, bloodstream-specific	Tb10.6k15.2290 (**2.4**, 28599)
UDP-Gal/GlcNAc-dependent glycosyltransferase	Tb927.3.5660 (2.0, 9638), Tb927.7.300 (**2.1**, 19406)
**Surface or secreted, transporters**	
64 kDa invariant surface glycoprotein	Tb927.5.1390 (**5.5**, 7559), Tb927.5.1430 (**3.6**, 6582), Tb927.5.1410* (**17.1**, 5352), Tb927.2.3270* (**6.9**, 18103), Tb927.2.3300* +3 (**5.6**, 13407), Tb927.2.332* (**8.1**, 14544), Tb11.47.0001* (**3.3**, 10214)
75 kDa invariant surface glycoprotein	Tb927.5.350 (2.4, 13044), Tb927.5.360-Tb927.5.360b (**4.7**, 17510), Tb927.5.370 (**6.2**, 11233), Tb927.5.400 (**4.2**, 13688), Tb927.5.390* (**5.9**, 17712)
acidic phosphatase (ISG65-like)	Tb927.5.630 (**3.7**, 29580)
flagellum-adhesion glycoprotein	Tb927.8.4060 (**4.5**, 11088)
glycophosphatidylinositol phospholipase	Tb927.2.600* (**3.4**, 17679)
haptoglobin-hemoglobin receptor	Tb927.6.440 (**4.0**, 15595)
ABC transporter	Tb11.02.3950 (2.1, 11483)
adenosine transporter	Tb927.5.286b (2.3, 9396), Tb927.2.6200 (**19.5**, 2940), Tb927.2.6220 (**20.7**, 3409), Tb927.2.6320 (**8.4**, 10291), Tb927.2.6280 (**20.1**, 7137), Tb927.2.6240 (**20.7**, 7104)
amino acid transporter	Tb927.4.4020 (**2.7**, 26379), Tb927.4.4860 (**2.3**, 9610)
aquaglyceroporin	Tb10.61.2650 (**4.2**, 28913)
glucose transporter	Tb10.6k15.2040 (**2.7**, 48061), Tb10.6k15.2030 (**4.4**, 23370)
glycerol uptake protein	Tb10.61.0380 (**2.3**, 11746)
major facilitator superfamily protein	Tb927.3.4070 (**5.3**, 24190), Tb927.3.4090 (**5.1**, 9918), Tb10.61.2750 (**2.5**, 8885)
UDP-galactose transporter	Tb927.4.1640 (2.3, 5225)
Vacuolar-type Ca2+-ATPase 2	Tb927.8.1200 (2.5, 14542)
**Other**	
kinesin	Tb10.61.1750 (**5.0**, 24612), Tb927.6.4390 (2.4, 12826), Tb927.7.4110 (2.5, 4714)
nucleolar protein	Tb09.160.1180 (2.3, 4877)
procyclin-associated gene	Tb10.70.1310 (**3.6**, 9070), Tb10.70.1300 (**9.9**, 7362), Tb11.01.6210 (**3.3**, 22849), Tb11.01.6220 (**4.1**, 10444)
retrotransposon hot spot protein	Tb09.v4.0013 (**7.2**, 3458), Tb09.244.2180 (2.8, 1283)
sarcoplasmic reticulum glycoprotein	Tb10.61.1710 (2.0, 9921)
VSG-related	Tb927.1.5060 (**2.5**, 5925), Tb927.1.5170 (**7.0**, 8241), Tb927.2.2060 (**3.1**, 8575), Tb927.3.1470-Tb927.3.1520 (**4.3**, 7116), Tb927.3.1500 (**33.1**, 13722), Tb927.3.1510 (**12.5**, 1450) Tb927.3.2540 (**3.3**, 2297), Tb927.3.5680 (**6.1**, 1903), Tb927.5.110 (**4.1**, 1468), Tb927.5.130 (**5.4**, 5216), Tb927.8.7300+1 (**3.7**, 16812), Tb09.160.5350 (**3.4**, 9129), Tb09.v1.0300+1 (**4.4**, 6354), Tb09.v1.0290+1 (**4.2**, 6386), Tb09.244.2330 (**5.2**, 3974), Tb09.244.2310 (**7.6**, 2584), Tb09.244.2280 (**3.1**, 881), Tb09.244.2240 (**5.1**, 2605), Tb09.244.2200 (**4.3**, 2404), Tb11.02.1566 (**8.0**, 12758)

^a^ESAGs, GRESAGS, *VSG*s are not included. A complete list of the genes is found in Additional file 3.

^b^Bold: gene was upregulated in all BF vs all PF conditions. Probe-sets detecting >1 gene indicated by "+", plus number of additional genes.

* marks known stage-regulated genes used in previous array study [12].

As can be seen in Figure 4A, categories of genes where cBF show higher expression than PF-log cells include *ESAG*s and *GRESAG*s, uncharacterized proteins bearing interaction motifs (such as zinc fingers and leucine-rich repeats), and known surface and secreted proteins (in part because there are multiple distinct genes in several surface protein families). However, it is also interesting that a larger number of genes upregulated in cBF encode proteins with hypothetical status (conserved or *T. brucei*-specific) that have signal sequences. This fits well with the finding that the secretory and endocytic systems are more active in BF than PF [18]. However, unlike Koumandou et al. [12], we did not find that mRNAs specifying proteins involved in secretory traffic were highly up-regulated in BF.

Categories with more representatives up-regulated in PF-log cells include those encoding mitochondrial proteins, metabolic proteins, and translation. It is known that the metabolism of PF (which can use both glucose and amino acids for energy metabolism) is more complex than BF (which are highly glycolytic) [19], presumably accounting for the more diverse set of metabolic genes up-regulated in PF-log cells. In PF, the mitochondrion becomes enlarged with more fully developed cristae and the respiratory chain is active [2]. These changes are reflected in the increased expression of a large number of genes (72) encoding products associated with the mitochondrion, including 27 that are of unknown function. In contrast, a single gene known to encode a mitochondrial protein is upregulated in cBF: the alternative oxidase. This oxidase is required for the glycerophosphate shuttle that allows glycolysis to continue [20].

The comparison of mRNA abundance between these two stages led to the identification of several groups of interesting genes. These include those encoding nucleoside transporters NT2-NT7 which reside in an array immediately adjacent to the sub-telomeric *VSG *cluster at the "right" end of chromosome 2. There they alternate with a set of iron-ascorbate oxidoreductase genes (see Tb927.2.6180, Tb927.2.6230, Tb927.2.6310) that have not been functionally characterized to our knowledge. The *NT *genes were reported to be more highly expressed in BF than PF forms, a finding which we also observe [21]. Interestingly, all of these oxidoreductase genes are also significantly more highly expressed in cBF than PF-log (3.3-13.2-fold, see Table 3). Three additional iron/ascorbate oxidoreductase genes are found on chromosomes 5, 7, and 9 -- these are each expressed to similar levels in cBF and PF-log. Thus, the chromosome 2 region represents a rare cluster of genes encoding similarly regulated mRNAs. Another interesting case is that of *VSP1*, an acidocalcisomal pyrophosphatase encoded by two tandemly linked genes (Tb11.02.4910 and Tb11.02.4930) with almost identical coding regions [22]. The array data show that the two genes are reciprocally regulated, which may potentially be traced to their divergent 3' UTRs.

### Comparison of gene expression in BF under different conditions

We compared the expression of all nuclear genes in slender BF isolated from infected animals with the expression in slender BF obtained by *in vitro *culture (cBF) and the expression in stumpy BF from animals. In comparison of cBF and slender BF, other than a few *VSG *genes, no gene showed a difference in expression that met our criteria of a 2-fold change and q-value < 5%. Additional file 5 lists those genes that showed more moderate (>1.5-fold) or less well-supported changes (q-value < 15). However, two *ISG64 *genes (Tb927.5.1390 and Tb927.5.1430) showed a slightly lower (1.6-1.8-fold), but high-confidence increase in signal in slender BF. A few other genes showed similar (1.5-1.9-fold) changes in expression, but had somewhat lower confidence (q-value = 7.9). These included a CAMK group protein kinase (Tb927.7.6580), a nucleoside phosphorylase (Tb927.8.4430), a tryparedoxin (Tb927.3.5090) and two proteins with unknown function that had higher signals in cBF, and a *GRESAG4 *that had a higher signal in slender BF. These data contrast with a previous microarray study examining 550 genes that found 35 were upregulated in cBF and 3 were upregulated in slender BF [12]. None of those 38 genes correspond to the few genes that we identified above. Two sets of genes that we identified as modestly upregulated were on the previous array, but these were not observed to be upregulated in that analysis. The lack of consistency between the two studies in this regard could arise from differences in the strains or conditions (*e.g*., medium, serum, use of intact *vs *immunocompromised animals). Nonetheless both studies do suggest that *in vitro *cultivation provides a reasonable model for analysis of most mRNAs in slender BF.

A comparison of the rapidly dividing slender BF with the non-dividing stumpy BF showed a total of 107 genes with at least a 2-fold change in signal in the arrays, not including *VSG*s. About twice as many genes were up-regulated in slender forms (Table 4) as were up-regulated in stumpy forms (Table 5). The most prominent categories of genes showing increased signals in slender forms are those that are related to the cytoskeleton, including the flagellum (Figure 4B). Many of these genes are annotated as hypothetical proteins, but they were detected in the flagellar proteome [23]. Non-dividing forms do not build new flagella or cytoskeleton. Additionally, several metabolic enzymes were up-regulated, predominantly those which are localized to the glycosome (a specialized peroxisome) or are involved in glycolysis. Conversely, the entire set of eight *ESAG9 *genes in the 927 strain were upregulated in stumpy BF (from 2-fold to 30-fold), as were two genes that are related to *ESAG9*. The function of *ESAG9 *is not known; it was originally described as a gene found in a *VSG *expression site (ES) in the closely related parasite *Trypanosoma equiperdum *[24]. At that time, the authors noted that a related *ESAG9 *was transcribed independently of the *VSG *ES. Seven of the eight annotated *ESAG9 *genes encode proteins with a predicted signal sequence, but none of these contain predicted transmembrane domains, suggesting the *ESAG9*s could encode a family of secreted proteins. The metabolic enzymes encoded by genes with higher mRNA levels in stumpy BF were predominantly mitochondrial, consistent with pre-adaptation for differentiation into insect forms. We also noted the increased mRNA for the *PAD1 *and *PAD2 *genes, which encode citrate transporters and were previously shown to be upregulated in stumpy forms of *T. brucei *strain EATRO 2340 [25].

**Table 4:** Genes showing increased expression in slender BF as compared to stumpy BF^a^

**description**	**Genes (fold change^a^, slender signal)**
adenylate kinase	Tb927.2.5660 (2.09, 12440)
alanine aminotransferase	Tb927.1.3950 (1.96, 10266)
amino acid transporter 8	Tb927.4.4860 (2.25, 11460)
cAMP-specific phosphodiesterase, PDEB1	Tb09.160.3590 (2.01, 6849)
clathrin heavy chain	Tb927.3.930 (2.12, 10381), Tb10.70.0830 (2.1, 13711), Tb10.70.1720 (1.90, 4250)
ESAG8	H25N7.22 (2.16, 15567)
flagellar axoneme protein PF16	Tb927.1.2670 (1.91, 15581)
flagellar component PACRGA	Tb927.3.2310 (1.74, 11680)
glycerol-3-phosphate dehydrogenase	Tb11.02.5280 (2.19, 28619)
haptoglobin-hemoglobin receptor	Tb927.6.440 (2.08, 14824)
histone H2A	Tb927.7.6360 (2.31, 4541)
hypothetical	Tb927.5.4010 (2.76, 18891), Tb05.5K5.220 (2.16, 8815), Tb927.8.7970 (2.65, 20178), Tb10.70.4020 (1.92, 7584), N19B2.190 (3.13, 7132)
hypothetical conserved	Tb927.1.4310 (2.39, 16196), Tb927.3.1910 (1.92, 6794), Tb927.4.2740 (2.36, 20142), Tb927.4.4580 (2.28, 9393), Tb927.4.4690 (1.93, 16059), Tb927.4.4700 (2.08, 10495), Tb927.5.2950 (1.83, 12001), Tb927.6.3180 (1.88, 12401), Tb927.7.6910 (2.36, 11887), Tb927.8.1550 (2.26, 16363), Tb927.8.3820 (2.09, 4078), Tb927.8.6660 (1.76, 26059), Tb10.389.0720 (2.70, 14817), Tb10.61.2210 (2.95, 9224), Tb10.61.3130 (2.03, 15809), Tb10.6k15.0710 (1.84, 8318), Tb10.70.4030 (1.96, 13839), Tb10.70.5560 (2.26, 11345), Tb10.70.7280 (1.76, 12000), Tb11.01.2700 (2.40, 6975), Tb11.01.4030 (1.73, 21437), Tb11.01.6470 (2.52, 17105), Tb11.02.0810 (1.91, 5728), Tb11.02.1660 (2.32, 10253), Tb11.02.4380 (1.97, 14847), Tb11.02.4400 (1.76, 21856)
hypothetical conserved, EF hand	Tb09.211.4820 (2.12, 6022)
hypothetical conserved, TPR repeats	Tb11.03.0240 (1.89, 18392)
hypothetical conserved, WD40 repeat	Tb09.211.4280 (1.99, 6137), Tb10.70.7320 (2.09, 12025), Tb11.02.5550 (2.01, 8496)
hypothetical conserved, zinc finger	Tb10.389.0740 (2.97, 15526), Tb11.01.8270 (1.99, 19491)
hypoxanthine-guanine phosphoribosyltransferase	Tb10.70.6660 (2.01, 12965)
inosine-adenosine-guanosine-nucleoside hydrolase	Tb927.3.2960 (2.06, 16716)
64 kDa invariant surface glycoprotein	Tb927.5.1410 (2.49, 8554), Tb927.5.1430 (2.02, 11573)
iron superoxide dismutase	Tb11.01.7550 (2.26, 11504)
leucine-rich repeat protein	Tb927.8.3790 (2.21, 6720)
mitochondrial carrier protein	Tb927.5.1550 (2.00, 5469)
mitochondrial DNA ligase homolog	Tb927.7.610 (1.96, 15064)
paraflagellar rod component	Tb11.01.5100 (2.08, 19728)
paraflagellar rod protein	Tb11.01.6740 (1.93, 17955)
phosphoglycerate kinase, glycosomal	Tb927.1.700 (2.19, 35215)
protein kinase	Tb11.01.4230 (2.55, 8847)
protein kinase, Aurora kinase AUK2	Tb927.3.3920 (1.97, 4982)
pumillio RNA binding protein PUF9	Tb927.1.2600 (2.39, 16018)
pyruvate kinase 1	Tb10.61.2680 (2.08, 29931)
RNA-binding protein	Tb11.01.3940 (2.28, 5191)
serine/threonine protein phosphatase	Tb05.5K5.30 (1.96, 5681)
SNF2 DNA repair protein	Tb927.7.4650 (1.96, 8894)

^a ^Fold-change as calculated by SAM analysis are shown; some genes showed 2-fold difference in Tukey means.

**Table 5:** Genes showing increased expression in stumpy BF as compared to slender BF

**description**	**Genes (fold change, stumpy signal)**
aquaporin 3	Tb927.6.1520 (1.67, 17418)
ESAG4	Tb11.03.0990 (2.65, 7195)
ESAG9	Tb927.1.5220 (4.68, 6148), Tb927.5.120 (3.86, 14115), Tb927.5.4620 (2.06, 3102), Tb927.7.170 (2.59, 24058), Tb09.160.5400 (6.63, 43669), Tb09.160.5430 (5.41, 6467), Tb09.v1.0330 (30,49, 25309), Tb11.1000 (4.20, 9262)
glutamate dehydrogenase	Tb09.160.4310 (2.43, 12969)
GPEET2 procyclin	Tb927.6.510 (8.51, 7966)
hypothetical conserved	Tb927.2.2140 (3.16, 3032), Tb927.6.4270 (1.98, 6654), Tb927.7.4270 (2.49, 13877), Tb09.160.0465 (2.61, 14430), Tb09.211.1620 (1.90, 8475), Tb09.v1.0490 (2.18, 28015), Tb10.389.1860 (2.22, 6264)
hypothetical conserved, aminotransferase domain	Tb927.4.2240 (2.16, 13035)
hypothetical conserved, ESAG9-like	Tb09.142.0370 (7.52, 7916), Tb09.142.0380 (2.63, 18446)
hypothetical protein	Tb09.v4.0151 (3.39, 27898), Tb10.70.2840 (2.57, 9259), Tb10.70.2850 (3.58, 3685)
major facilitator superfamily protein, PAD2	Tb927.7.5940 (2.04, 36576)
mitochondrial processing peptidase alpha subunit	Tb11.02.1480 (2.01, 9711)
2-oxoglutarate dehydrogenase E2 component	Tb11.01.3550 (2.29, 11013)
protein phosphatase with EF-Hand domains	Tb927.8.1130 (2.24, 9691)
purine nucleoside transporter	Tb09.160.5480 (2.34, 30007)
pyruvate dehydrogenase complex E3 binding protein	Tb10.70.5380 (2.10, 13843)
RNA-binding protein RBP5	Tb11.01.3915 (2.85, 9818)
serine/threonine-protein kinase NrkA	Tb927.8.6930 (1.78, 30611)
succinyl-coA:3-ketoacid coA transferase mitochondrial	Tb11.02.0290 (1.86, 22800)
transketolase	Tb927.8.6170 (2.16, 4917)
transporter	Tb10.61.2747 (1.79, 9346)
VSG-related VR2.1	Tb11.01.4560 (2.48, 18586)

^a ^Fold-change as calculated by SAM analysis is shown; some genes showed 2-fold difference in Tukey means.

### Comparison of gene expression of PF in different conditions

Unlike cBF, *in vitro *cultured PF can be grown to stationary phase where they can persist for several days as viable cultures. Thus, we could directly compare the abundance of mRNAs in actively replicating (PF-log) and non-dividing (PF-stat) cells. A total of 895 genes showed differential expression (see Additional file 6), many more than the 107 genes differentially regulated between with the slender (log) versus stumpy (stationary) BF. About three times as many genes were up-regulated in PF-log as compared to PF-stat. As shown in Figure 4C, this increase was reflected across almost all categories of genes, except for proteins categorized as unknown (both conserved and *T. brucei*-specific). The most skewed group was genes annotated as encoding hypothetical proteins (conserved or *T. brucei*-specific) that have predicted transmembrane domains -- many more such genes were upregulated in stationary phase than in log phase. For proteins with ascribed function, those associated with protein phosphorylation/dephosphorylation were enriched in stationary phase. As discussed above, some of changes in PF-stat may reflect the decrease in cellular growth functions, or perhaps preparation for development to epimastigotes. It is also possible that some transcripts with higher signals in PF-stat are simply those that decay most slowly.

### Genes encoded by the mitochondrial genome

Several genes on the mitochondrial maxicircle genome are extensively remodeled by RNA editing to yield transcripts encoding components of mitochondrial respiratory complexes. Only 15 mitochondrial probe-sets could be designed (see Additional file 7). Four corresponded to both edited and unedited sequences, and six to never-edited sequences, including the two rRNAs. Three corresponded to edited sequences, two of which had corresponding unedited probe-sets. From this limited set, a few trends could be observed, which were compatible with prior literature [26-28]. For example, 12S and 9S rRNA, cytochrome b, cytochrome oxidase subunit I, and cytochrome oxidase subunit II (edited plus unedited) transcripts all increased in stumpy BF and further increased in PF-log, although some did not reach statistical significance until PF-log phase. Somewhat surprisingly, ATP synthase subunit 6 (edited), NADH dehydrogenase subunit 5, NADH dehydrogenase subunit 7 (edited) and NADH dehydrogenase subunit 8 (edited) all showed increased mRNA levels in stumpy BF, but decreased in PF-log. Unexpectedly, many of the signals reached their maximum in PF-stat. This could reflect a potential differential stability as compared to the nuclearly-encoded transcripts under conditions of growth arrest, and would be highlighted by the normalization procedure.

### Gene families

**Figure 5:**
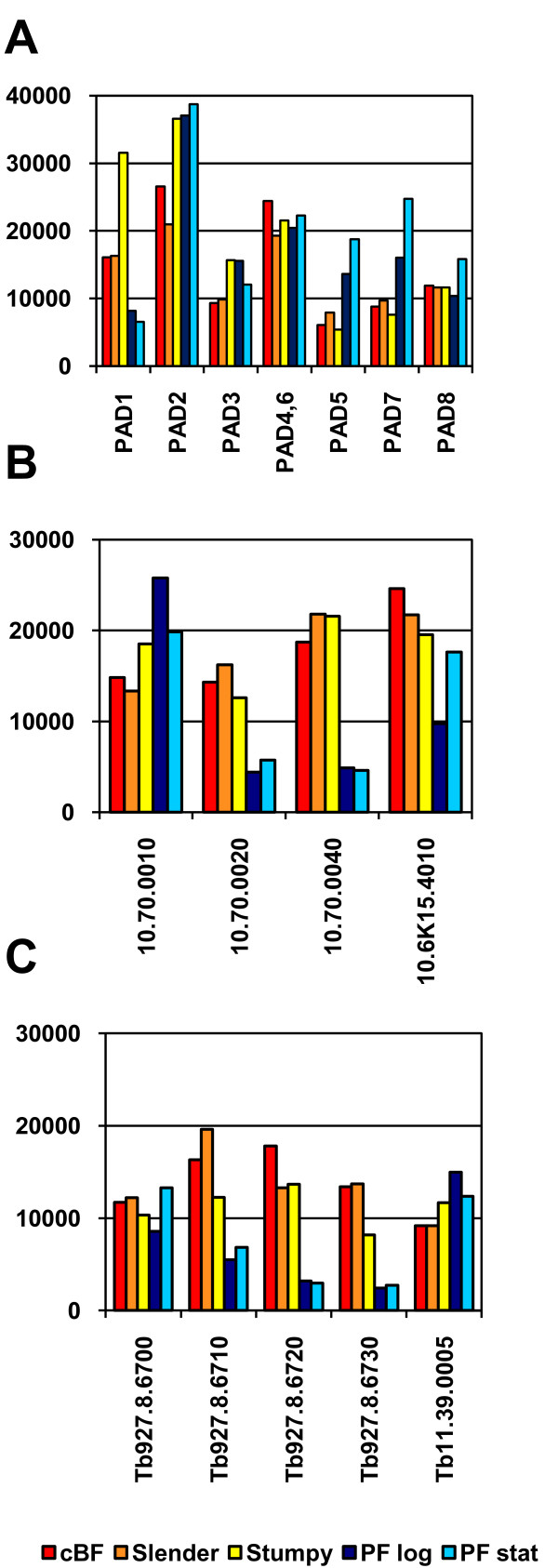
**Differential expression of tandemly arrayed genes**. **A**. The PAD gene array of carboxylate transporter proteins (*PAD1*, Tb9277.5930, *PAD2*, Tb927.7.5940, *PAD3*, Tb927.7.5950, *PAD4*, Tb927.7.5960, *PAD5*, Tb927.7.5970, *PAD6*, Tb927.7.5980, *PAD7*, Tb927.7.5990, *PAD8*, Tb927.7.6000). Not shown is the unlinked PAD-like gene, Tb927.8.1650, which is expressed constitutively to low levels. **B**. A cluster of four genes encoding proteins of a major facilitator family. **C**. A cluster of genes with five to six transmembrane domains. Four genes are tandemly linked on chromosome 10 and the fifth, more divergent gene (Tb11.39.0005) is on chromosome 11.

We noted several tandem arrays of gene families containing non-identical genes that were differentially regulated. Three families encoding proteins with multiple transmembrane domains are depicted in Figure 5. The first cluster (Figure 5A) of genes are those in the recently described *PAD *array of carboxylate transporters [25]. In contrast to *PAD1 *and *PAD2*, which are induced in stumpy BF [25], the other members of this gene family are either constitutively expressed at the mRNA level or more highly expressed in PF. *PAD5 *and *PAD7 *show an increase in expression from stumpy forms to PF-log and even higher expression in PF-stat. *PAD8 *showed similar expression in all conditions except in PF-stat, which was ~1.5-fold increased over PF-log (q-value = 4.89). Figure 5B shows an unrelated gene family on chromosome 10 that also encodes major facilitator proteins. These four genes show a high level of conservation with one another, with long stretches of amino acid identity. Two are most highly expressed in BF, whereas the other two show a more complex pattern of regulation. The final set of genes (Figure 5C) encodes a set of related proteins predicted to have four to five transmembrane domains, four of these genes are tandemly arrayed on chromosome 8. Here the mRNA abundances of the three most closely related genes are higher in the BF samples. In contrast, the first gene in the array and another more divergent, unlinked gene on chromosome 11 do not show this pattern, and have similar or higher expression in PF.

### VSGs and ESAGs

The *T. brucei *strain 927 genome contains approximately 1600 *VSG *genes (or pseudogenes), but each BF trypanosome expresses only a single VSG, which covers the surface of the parasite in a dense coat. Although *T. brucei *possesses ~20 *VSG *ESs (located at telomeres of megabase- and intermediate-sized chromosomes) [29], the expressed *VSG *gene encoding the surface coat protein is located in the sole active ES. In BF, transcription initiates in all ESs, but attenuates rapidly in the inactive ESs, never reaching the downstream genes including the resident *VSG *[30]. Similarly, transcription of ESs initiates in PF, but transcript elongation is minimal [31]. A relatively small number of apparently functional *VSG *genes exist on the 11 megabase-sized chromosomes in *T. brucei*. The minichromosomes also contain a reservoir of apparently functional *VSG *genes, but only a few have been sequenced. In contrast, most *VSG *genes reside in sub-telomeric arrays that are comprised of pseudogenes (which were not included on these microarrays) and atypical *VSG *genes, which encode proteins that are neither clearly pseudogenes nor clearly functional [11]. The pseudogenes provide the fuel for generating novel *VSG *genes by mosaic gene conversion during antigenic variation, particularly later in infection [32]. The VSG-related *VR *genes are located not in the telomeric ESs or sub-telomeric arrays, but rather typically reside in chromosome-internal strand-switch regions and lack the 70-bp repeats typically found upstream of *VSG *genes [11,32]. The telomeric ESs and sub-telomeric *VSG *arrays also contain hundreds of *ESAG*s, many of which are pseudogenes. However, a number of genes related to *ESAG*s (*GRESAG*s) have chromosomal-internal location (the nomenclature discriminating *ESAG*s and *GRESAG*s was not consistently applied as genes were named).

**Figure 6:**
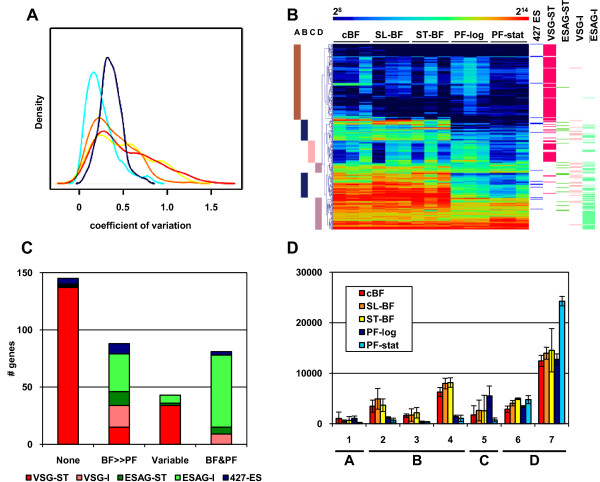
**Cluster analysis of *ESAG *and *VSG *gene expression**. **A**. Signals from probes detecting *VSG/VR *genes. Density plot of gene-level coefficient of variation for *VSG *genes, defined as the standard deviation across the three biological replicates divided by the mean value of the replicates. The lines are cBF (red), slender BF (orange), stumpy BF (yellow), PF-log (dark blue), and PF-stationary (light blue). **B**. Heatmap of the *ESAG/GRESAG *and *VSG*/*VR *genes after hierarchical clustering of the individual sample log_2_-transformed raw expression values. The clusters were pooled into four different expression patterns as shown on left. At right the genomic location of genes is shown: 427-ES, those detected by probe-sets corresponding to sequenced 427 strain *VSG *expression sites; I, chromosome internal; ST, sub-telomeric as defined by occurring distal to the first or last strand-switch region on the chromosome. **C**. Representation of each gene class in the four clusters, classified as either sub-telomeric (VSG-ST, ESAG-ST) or chromosome internal (ESAG-I, *VSG*-I). Some of the genes derived from the 427 ESs last are likely not present in strain 927, while the remainder reside in regions not sequenced in strain 927 such as the intermediate or minichromosomes and the telomeres. **D**. Examples of *VSG*/*VR *genes from the four groups. The mean and standard deviation of the individual samples in each biological condition is shown. Genes are: group A (**1**, *VSG *Tb11.57.0024), group B (**2**, *VSG *Tb09.v4.0012, **3**, *VR10 *Tb927.5.110, **4**, *VR16 *Tb09.v1.0300), group C (**5**, unclassified *VSG *Tb05.5K5.440), and group D (**6**, atypical *VSG *Tb927.4.5400, **7**, *VR *Tb927.5.291b).

The microarray design used in this study, contained probes for 74 *VSG*s, 70 atypical *VSG*s, and 46 *VSG*s that were unclassified on VSGdb [33]; 21 sub-telomeric *ESAG*s, 104 chromosome-internal *ESAG*s and *GRESAG*s, as well as 17 *ESAG*s from three *T. brucei *strain 427 ESs (no *T. brucei *strain 927 ESs have been annotated to date). This *VSG *and *ESAG *subset of genes was represented by a total of 357 probe-sets. Even though individual parasites express only one ES (containing a single *VSG *and ~10 *ESAG*s) at a time, since the parasites have been maintained without regard for antigenic type, we expected that there would be diverse set of *VSG *genes showing some expression at the population level. In addition, we expected that expression of these *VSG*s and *ESAG*s would vary between biological replicates, and indeed, a subset of *VSG *and *ESAG*s showed considerable variation in BF, but not PF (Figure 6A), probably reflecting antigenic variation within these populations. Thus, subsequent analyses were carried out on the 15 individual samples rather than on the mean of the biological conditions (see Additional file 8 for gene level data).

Hierarchical clustering of the 357 probe-sets (after log_2_-transformation of the normalized expression values) allowed us to define four distinct patterns of *VSG *gene and *ESAG *expression (marked A-D in Figure 6B). Interestingly, the distribution of *VSG *genes and *ESAG*s from different genomic locations within each group differed markedly (see Figure 6B and 6C). Group A contained a large number (137) of *VSG*s not expressed in any sample, or only at low levels in some BF samples, exemplified by gene 1 in Figure 6D. All these genes were located within sub-telomeric clusters and were likely not transcribed at any stages, except when translocated to the active expression site in small sub-populations of BFs. This group also included five *ESAG*s from *T. brucei *427 ESs that presumably either reside in inactive expression sites or are not present in *T. brucei *927.

A second group (B) contained 34 *VSG *genes and 54 *ESAG*s, which were expressed at substantially higher (but still relatively moderate) levels in BF and generally low levels in PF. Many of these showed variable expression levels in different biological replicates of the BF samples, indicative of expression from active ESs in sub-populations of BF. This group contained *VSG *and *VR *genes from sub-telomeric clusters (genes 2 and 3, in Figure 6D), as well as from chromosomal-internal locations (mostly *VR*s, *e.g*. gene 4). It also contained *ESAG*s and *GRESAG*s from the 427 ES, sub-telomeric clusters and chromosomal-internal loci. Of particular interest are several *ESAG9 *genes that are up-regulated only in stumpy BF (as discussed above). While this group of genes has many of the hallmarks of canonical *VSG/ESAG *expression from ESs, it should be noted that in many cases their signal levels in PF were substantially above background; suggesting that the genes are actively transcribed in PF, but the mRNAs are less stable than in BF.

Unexpectedly, a group (C) of 34 sub-telomeric *VSG *and nine *ESAG *genes showed variable expression levels in both BF and PF. The function of these *VSG *genes is unclear, since they appear to encode both typical and atypical VSGs. In particular, one group of four tandemly-linked *VSG *genes from an allele-specific region of chromosome 5 showed highest expression in PF-log cells (see gene 5 in Figure 6D). Group C contains both sub-telomeric and internal genes encoding ESAGs 2, 3, 5, and 11. Interestingly, several *ESAG11*-related genes and a *VR *are located in a tandem array on chromosome 4, where they are interspersed with genes encoding hypothetical proteins. Since the hypothetical proteins show very similar expression patterns to the adjacent *ESAG11*-related genes, at least some may simply represent 3' UTRs of the neighboring genes. Interestingly, this cluster is located between rRNA and tRNA gene clusters, and would not be expected to be transcribed, since it appears to lack the modified chromatin found at typical RNA polymerase II transcription initiation sites [15,16]. The signal levels for all these genes is modest (<3000), and are lowest in stationary phase, suggesting that they may merely represent increased "background" transcription due their proximity to the actively transcribed RNA genes. However, this does not rule out functionality of this set of putative genes.

The final group (D) of *VSG *and *ESAG *genes were expressed in all life cycle stages. All nine *VSG*/*VR *genes in this group are located in chromosomal-internal loci: four are annotated as *VR*s, four are atypical *VSG*s and one is uncategorized. mRNA for several of the *VR *genes has previously been detected in PF using PCR [32]. One of the *VR*s shows highest expression in PF-stat (gene 7 in Figure 6D). Three of the atypical *VSG *genes show similar expression in all stages (e.g., gene 6 in Figure 6D). These genes, Tb927.4.5400, Tb927.4.5420 and Tb927.4.5430, along with a 4^th ^gene identical to Tb927.4.5420, are tandemly-linked to form a small cluster just 5' (and on the opposite strand) to the sub-telomeric *VSG *cluster at the "right" end of chromosome 4. Interestingly, this cluster of genes is immediately downstream of a convergent strand-switch region that appears to contain an RNA polymerase transcription initiation site in both BF and PF [16]. A large number of *ESAG*s are also expressed in all life cycle stages; of these most are chromosomal-internal *GRESAG4 *genes that have been shown previously to be expressed in PF [34]. However, two *ESAG4*s and an *ESAG7 *from the 427 ES show this expression pattern, as do six sub-telomeric *ESAG*s (two encode ESAG3, two ESAG5, one ESAG4 and one ESAG9-like). The functional significance of their expression in PF is unknown.

Of the 215 *VSG *genes examined, 43 showed expression levels above the bottom quartile (~4500) of all genes in at least one BF sample. These included 10 classified as encoding functional VSGs, and eight that were unclassified, but also included eight encoding atypical VSGs and 17 *VR*s. From these data it is apparent that at least some atypical *VSG*s are expressed and hence likely to be functional. Indeed a query on GeneDB for *VSGs *annotated as being detected in proteomic analysis of BF [35] yielded seven genes, two of which are atypical VSGs. Only nine of the 43 *VSG *genes noted above were expressed below the 5^th ^percentile (~1300) in PF. These included five encoding typical VSGs (and two that were uncharacterized), but one gene encoded an atypical VSG, and one *VR *gene also had this expression pattern. Thus, these data suggest that the functional diversity of *VSG *and *VR *genes is likely more complex that currently appreciated.

## Conclusion

The results obtained in our study for genes previously shown to be differentially expressed in BF *versus *PF, including those used as controls in a previous microarray analysis [12], allow us to critically assess the validity of our analysis. As expected, mRNAs corresponding to pyruvate kinase 1, metacaspase 3, isoforms of ISG64, ISG65, and ISG75, VSG glycophosphatidyl inositol phospholipase, major surface protease MSP (also known as GP63) isoforms A and C, hexokinase I and phosphoglycerate kinase isoform C all showed increased levels in cBF; while EP2 and EP-3-2 procyclins, a trans-sialidase, phosphoenolpyruvate carboxykinase, cysteine-rich acidic integral membrane protein (CRAM), a flagellar adhesion protein, corset protein 17, and CAP5.5 [36] all showed increased signal in PF-log samples, although the changes generally were less dramatic than those seen on Northern blots and in the previous microarray analyses. Seven genes previously used as differentially expressed controls in [12] showed changes less than 2-fold in our hands. Four showed more modest changes and one (encoding histone H3) did not change between cBF and PF-log, although it was down-regulated in PF-stat. Finally, recent data suggest that two of those genes may not be regulated between these stages [36,37]. Thus, there is good agreement between the current results and previous work. Since eight probes were used for most genes in our study, it is likely that any changes we see are highly reliable. The somewhat muted differences could be due to a lower sensitivity of the microarrays or to strain variations [38].

The data we obtained shows that despite the lack of transcriptional control for most genes, gene expression is finely tuned during *T. brucei *development. While most of changes in mRNA abundance were relatively modest, some varied by 10- to 100-fold, and over one-fourth changed at least 2-fold between the different conditions. Many of these changes were easy to reconcile with known changes in parasite biology: the transition from dividing to non-dividing forms affects genetic functions such as transcription and translation and the transition from mammalian to insect stages affects metabolism, surface proteins and transporters. A moderate number of genes encoding proteins with functions associated with RNA were differentially expressed, perhaps reflecting the important role these play in post-transcriptional regulation of gene expression. It is perhaps surprising that relatively few genes encoding protein or lipid kinases and phosphatases were highly regulated, although this may reflect a predominant use of phosphorylation pathways to modulate the activities of these enzymes themselves.

A recently published set of analyses of the transcriptomes of the four developmental stages of *Trypanosoma cruzi *indicated that up to 50% of genes show a statistically significant difference in mRNA levels during parasite development [39], a proportion considerably higher than that which we observed in *T. brucei*. It is likely that as other stages of the *T. brucei *life cycle are examined, the proportion of genes determined to show significant regulation during development will rise. Our findings can also be compared to studies in related parasite *Leishmania *where ~3-10% of the genes showed changes of at least 2-fold between rapidly-growing procyclic promastigotes, non-dividing metacyclic promastigotes, and the more slowly-growing amastigote forms [40-45]. While the studies in *Leishmania *identified a heterogeneous, but overlapping, set of mRNAs showing regulation in abundance, several similarities with the present study emerge. These include down-regulation of genes encoding translational machinery (including tRNAs) in slower growing stages, as well as substantial changes in mRNA levels for many metabolic enzymes.

Interestingly, our results show very little difference between cultured and animal-derived *T. brucei *BF. Other studies using stresses such as exposure to tunicamycin or reducing agents, reduced serum or genetic manipulation of specific genes showed few changes in RNA abundances, suggesting that BF parasites have little ability to alter their transcriptome following unexpected environmental changes [12]. This contrasts with some reports of substantial differences between axenic and animal-derived amastigotes of *Leishmania *[46]. It remains to be seen whether this difference is due to the extracellular nature of *T. brucei*, methodological difficulties of extracting amastigotes from macrophages, or novel environmental cues detected by the *Leishmania *parasites.

Use of the Nimblegen arrays also allowed us to provide an assessment of the relative abundance of transcripts within a biological condition, over at least two orders of magnitude. While many of the highly expressed transcripts encode well-characterized proteins, there still remain many that have received little or no attention. The data presented here provide an important foundation for researchers interested in elucidating the unusual biology of the parasite or developing new interventions to combat the lethal disease they cause. It is now important to further understand the mechanisms involving regulation of translation, protein activity, and protein turnover to realize the full extent of developmental regulation in *T. brucei*. This is underscored by recent work comparing changes in the transcriptome and proteome during *Leishmania *differentiation, which suggests that there is a relatively poor correlation between the two and that translation and protein stability play important roles in regulation of gene expression in trypanosomatids [47](Lahav et al., manuscript in preparation). However, work in *T. cruzi *suggests a more robust relationship [39], perhaps pointing to different modes of gene regulation between the *Leishmania *and *Trypanosoma *genera.

## Methods

### Parasites and RNA preparation

The pleiomorphic *T. brucei *strain TREU927/4 was used for all analyses. All procedures involving vertebrate animals followed protocols approved by the institutional IACUC. For isolation of slender BF and stumpy BF, Wistar rats (retired-male breeders) were immunosuppressed with 750 rads. Animals were immediately infected with 1-2 × 10^8 ^parasites by intraperitoneal injection and the parasitemia was monitored daily beginning on day 2 post-infection. For slender BF populations, animals were sacrificed for harvest on day 4, when the parasitemia was still increasing. For stumpy BF populations, animals were sacrificed when the morphologically stumpy population in the blood smear exceeded 70%. Blood from infected animals was harvested with 10 mg/ml heparin in phosphate-buffered saline (PBS) with 10 mM glucose. The samples were then centrifuged at 900 × g for 10 min and the buffy coat containing the parasites extracted. To remove the remaining red blood cells, the buffy coat was loaded onto to a DE53 (Schleicher and Schuell) column that had been equilibrated with room temperature PBS plus glucose, hypoxanthine (0.35 μg/ml) and L-cysteine (80 μg/ml). Parasites were eluted from the column with the same buffer and pelleted at 900 × g for 10 min. RNA was immediately isolated as described below.

Stumpy BF begin to transform to PF quickly and synchronously, as determined by expression of EP procyclin on their surface four hours after induction, while neither slender or intermediate BF will express EP procyclin within this time frame [5,48]. To assess EP procyclin expression, ~5 × 10^7 ^purified parasites were pelleted and resuspended in DTM medium [49] with 6 mM freshly prepared cis-aconitate and incubated for 4 hours at 25°C. Washed cells were fixed in PBS with 3.7% formaldehyde for 10 min at room temperature. Fixed cells were washed once with PBS and stored in 100 μl PBS at 4°C for up to 24 hours. Cells were spotted on poly-L-lysine coated slides and, after blocking with 10% goat serum, surface-localized procyclin detected with monoclonal antibodies with a mixture of two monoclonal antibodies against EP procyclin (antibody 16, directed against the glutamine-proline repeats of EP procyclin and antibody 418, also directed against EP procyclin) [50] at a 1:500 dilution for 1 hour. Slides were processed and cells visualized as described [51]. PF strain 29.13 and cBF single marker strain were similarly stained as positive and negative controls respectively. For all counts, at least one hundred cells were scored.

HMI-9 medium [52] with 10% fetal bovine serum was used for continuous culture of cBF. The three biological replicates were prepared several months apart. Parasites were seeded into 400 ml of media and grown to ~8 × 10^5 ^cells/ml, to obtain a log-phase population. Cells were pelleted and RNA isolated as described below.

### Generation of PF from stumpy BF

Stumpy BF obtained from an infected rat by cardiac puncture were diluted in SDM-79 medium [53] containing 15% fetal bovine serum. The sample was centrifuged at 200 × g for 10 min to pellet the majority of the blood cells, while leaving the majority of parasites in the supernatant. The supernatant was then transferred to a flask and cis-aconitate was added to 6 mM to induce differentiation to PF at 26°C. After 2 hours, most of the remaining blood cells had settled to the bottom and the upper phase of the medium was transferred to a fresh flask and allowed to incubate overnight. The next day, the cells were again transferred to a fresh flask and the cultures were monitored daily until parasites started growing. The growing PF expressed EP procyclin in the immunostaining assay described above. For PF-log samples, cultures were grown to 4 × 10^6 ^cells/ml, split 1:1, and allowed to grow an additional 24 hours before harvest at densities of 6-7 × 10^6 ^cells/ml (see Additional File 1). For PF-stat samples, cultures were started at 10^6 ^cells/ml and cell number and integrity was monitored daily beginning day 3. RNA was isolated on day 5, the first day without any increase in cell density (at about 7.5 × 10^7 ^cells/ml). The next day cultures showed a significant increase in the proportion of dead cells.

### Isolation of RNA

Cell pellets were resuspended in 1-2 ml TRIzol (Invitrogen), with a maximum of 5 × 10^8 ^cells per ml TRIzol. RNA was then isolated according to the manufacturer's directions. The quality of the RNA was verified by running on an Agilent 2100 Bioanalyzer. RNA was sent to Nimblegen for cDNA synthesis using oligo-dT priming, labeling and hybridization to oligonucleotide arrays. Because of the method of priming, the expression levels of RNAs, mRNAs and mitochondrially-encoded transcripts cannot be directly compared. However, both edited and non-edited mitochondrial transcripts of *T. brucei *have poly(A) tails [54], although the tails are longer for edited transcripts [55]. Because transcripts from genes that do not encode proteins (*e.g*. snRNAs, tRNAs and snoRNAs) are usually not polyadenylated, signals corresponding to most of these genes were low, and probably resulted from limited priming from oligoA tracts.

### Array design

The nucleotide sequence of 8530 protein-coding sequences (CDS) and 559 RNA genes predicted from the *T. brucei *927 genome assembly (version 4) was obtained from the GeneDB ftp site (genes annotated as "hypothetical unlikely", pseudogenes, or residing on intermediate-sized chromosomes were excluded) and provided to Nimblegen for array design, aiming for eight probes per CDS and three probes per RNA (probes were 60 bp). Since the strain 927 telomeres were not sequenced, we also included 50 CDSs predicted from three BACs from strain 427 telomeric expression sites. In additional, the two RNA genes and 18 CDSs from maxicircle (mitochondrial) DNA [GenBank:M94286] and the corresponding edited transcripts were included in the array design.

To identify probes that would likely hybridize with more than one gene, we utilized CROSS_MATCH[56]. All probes to the CDS and RNA dataset above, as well as the entire *T. brucei *927 genome sequence, were tested using a min_match of 35 and min_score of 55. Gene-specific probes were defined as those that had no more than one high-quality match (*i.e*. 35 contiguous perfectly matched nucleotides with no more than 5 mismatches per probe) against the entire genome. All probes that were not specific to a single gene (or set of near-identical genes) were removed from subsequent analyses. This resulted in 8004 probe-sets corresponding to nuclear protein-coding genes, 106 corresponding to nuclear RNA genes, 14 corresponding to maxicircle protein-coding genes and 2 corresponding to maxicircle rRNA genes (see Additional file 2). Of these, 345 probe-sets detected more than one identical (or near-identical) protein-coding gene; a few of these probe-sets detected a pseudogene or "hypothetical, unlikely" gene in addition to the original gene of interest. This was most common for *VSG*s. For simplicity, in this communication we refer to all genes detected by a probe-set as "a gene". In the final analyses, 93% of the CDS probe-sets contained eight probes, while 54% of the RNA genes were represented by three probes. Each 60 bp probe was randomly assigned to three different spots on the array.

Only 409 *T. brucei *genes could not be analyzed (see Additional file 9); these included mosaic genes, as well as those for which probes could not be designed or for which all probes matched other (mostly unannotated) regions of the genome. Most corresponded to RNA genes, leaving only 124 protein-coding genes without a corresponding probe-set.

### Analysis of microarray data

The probe intensity signals from the 15 microarray chips (three biological replicates by five conditions), as provided by Nimblegen, were subjected to quantile normalization to account for non-biological signal intensity variation across the chips [57]. This data has been deposited with the Gene Expression Omnibus (GEO) database (Accession no.: GSE18049), and has also been provided to TriTrypDB  for public access. A single gene-level value for each biological replicate was determined by calculating a weighted mean of all probes in each probe-set using the Tukey biweight formula, which minimizes the influence of outlier values. The statistical significance of signal changes between samples was assessed by either pair-wise or multiclass tests from the Significance Analysis of Microarrays (SAM) software package [58], as appropriate, using all three biological replicates for each condition. We set the q-value to 5, yielding a false discovery rate of 5% for the set of genes identified. There was good agreement between genes showing a 2-fold or greater change assessed by the SAM-calculated mean and those identified using a Tukey biweight mean of the biological replicates. Pair-wise analyses considered both values, accepting all genes with at least a 2-fold change by one method and at least a 1.7-fold change by the other method.

Several different cluster analyses were performed using the MeV component of the TM4 software package [59]. The three biological replicates were combined by Tukey biweight function to give a single gene-level value for each of the five conditions and hierarchical clustering [60] was carried out on all 8110 probe-sets using the following parameters: Gene Tree selection only; Gene Leaf Order optimization; Euclidean Distance metric and complete linkage clustering. In order to focus on genes showing the most variation between biological conditions, we selected those in which at least one condition showed >2-fold deviation from the mean of all five biological conditions and q-value of < 5% in multi-class SAM analysis, and excluded all *VSG *and *VSG*-related (*VR*) genes and *ESAG*/*GRESAG*s. The log_2_-transformed fold-values from the resultant 534 probe-sets were K-median clustered [61] by genes only using the Euclidian distance and 50 iterations, with hierarchical clustering of genes within the nine clusters using the parameters described above. The 357 probe-sets representing *VSG*/*VR *genes and *ESAG/GRESAG*s were separately analyzed by hierarchical clustering of values (log_2_-transformed Tukey biweight means) for the individual biological samples for all five different conditions, using the same parameters as above. The HCL tree was split into six clusters and two of these clusters further divided two sub-clusters. The clusters and sub-clusters were then combined into four different groups based on similarities in expression pattern.

All protein-coding genes were manually assigned (based on their GeneDB product description and literature review) into 17 functional categories that are particularly relevant to trypanosomatid parasite biology. These included:

- DNA-associated (histone-related or histone modifying, proteins involved in DNA replication, repair, or chromatin remodeling, proteins with DNA binding motifs, and nucleases)

- ESAGs/GRESAGs

- Interacting (proteins with interaction motifs such as zinc fingers, leucine rich repeats, PX domains, PH domains, and WD40 domains but with no other attributed function)

- Metabolism (metabolic enzymes)

- Oxidative stress (peroxidases, peroxiredoxins, superoxide dismutases, trypanothione metabolism)

- Phosphorylation (protein and lipid kinases and phosphatases)

- Protease-related (peptidases, proteases, protease-related, and ubiquitin pathway)

- Protein folding (chaperones, proteins involved in protein folding or unfolding)

- Protein transport/modification (proteins mediating protein trafficking within the cell including the endomembrane system and organelles or involved in modification of proteins within the endomembrane system such as glycosylation)

- RNA-associated (RNA binding proteins, helicases, nucleases)

- Transcription (RNA polymerase subunits and transcription factors)

- Translation (proteins involved in ribosome biogenesis or translation including nucleolar proteins, ribosomal proteins, tRNA synthetases and tRNA modifying enzymes)

- Transporter (membrane proteins transporting small molecules)

- *VSG/VR*

- Other (any protein with functional annotation other than those above, plus those with known subcellular location discussed below)

- Unknown, conserved (those annotated in GeneDB as "hypothetical protein, conserved and not assigned to any of the categories above)

- Unknown, *T. brucei*-specific (those annotated in GeneDB as "hypothetical protein" and not assigned to any of the categories above)

A subset of proteins (~900) were assigned to subcellular location categories on the basis of GeneDB annotation, literature surveys, or proteomic analyses. Assignment to the cytoskeleton included those identified in the flagellar proteome [13], while those with mitochondrial location included those identified in the high-confidence mitochondrial proteome [14]. Other assignments were less exhaustive and focused on differentially expressed genes. Surface/secreted proteins included those known to be secreted or localized to the parasite surface based on experimental analysis (ESAGs, GRESAGs, transporters, and VSGs were not included). Both functional categories and location were used to group genes for different analyses, as indicated in the Figure Legends. Additional file 2 provides gene-level information on gene categories, clusters, probes and signals, and SAM analyses for all nuclear genes studied.

## List of abbreviations

BF: bloodstream forms; cBF: *in vitro *cultured bloodstream forms; CDS: protein coding sequences; ES: expression site; ESAG: expression site-associated gene; GRESAG: gene related to expression site associated-genes; ISG: invariant surface glycoprotein; PBS: phosphate-buffered saline; PF: *in vitro *cultured procyclic forms; PF-stat: *in vitro *cultured procyclic forms in stationary phase; SAM: significance analysis of microarrays; UTR: untranslated regions; VR: VSG-related; VSG: variant surface glycoprotein.

## Authors' contributions

BJ was responsible for the preparation of RNA, contributed to the analysis of gene function and expression, and assisted in preparation of the manuscript. CK was responsible for the preparation of the stage-specific trypanosome populations. DS designed the microarray and performed statistical analyses. MP conceived and coordinated the study, contributed to the analysis of gene function and expression, and participated in drafting the manuscript. PJM assisted in study design, contributed to the analysis of gene function and expression, and participated in drafting the manuscript. All authors read and approved the final manuscript.

## Supplementary Material

Additional file 1 **Growth curve for PF**. Growth curve showing cell densities at time of harvest of procyclic forms. Click here for file

Additional file 2 **Microarray gene level data**. Expression data and attributes for all nuclear genes on the array (data) plus key describing column headers (key). Click here for file

Additional file 3 **File 4 Most highly regulated genes**. Listing of the most highly regulated nuclear genes, including changes in gene expression compared to the mean expression level across all conditions (data), plus key describing column headings (key). Click here for file

Additional file 4 **cBF vs PF log**. Genes differentially expressed in cBF versus PF-log Click here for file

Additional file 5 **Slender BF vs cBF**. Genes differentially expressed in cBF versus slender BF Click here for file

Additional file 6 **PF-log vs PF-stat**. Genes differentially expressed in PF-log versus PF-stat Click here for file

Additional file 7 **Maxicircle genes**. Expression data for mitochondrial maxicircle genes Click here for file

Additional file 8 **VSGs and ESAGs**. Individual sample expression data for *VSG *and *ESAG *genes Click here for file

Additional file 9 **Genes not on array**. Listing of genes for which array data was not obtained (data) plus key describing column headers (key). Click here for file
