# Supplementary material for: Widespread variation in transcript abundance within and across developmental stages of Trypanosoma brucei
Source: BMC Genomics. 2009 Oct 19;10:482. doi: 10.1186/1471-2164-10-482 (PMC2771046; doi:10.1186/1471-2164-10-482)

Additional File 1. Jensen et al.

Growth curve for procyclic forms grown in culture. Stationary-phase cultures were harvested on day 5 for RNA preparations. Curves 1-3 correspond to the separate replicates. Log-phase cultures were harvested on day 3 of similar cultures.

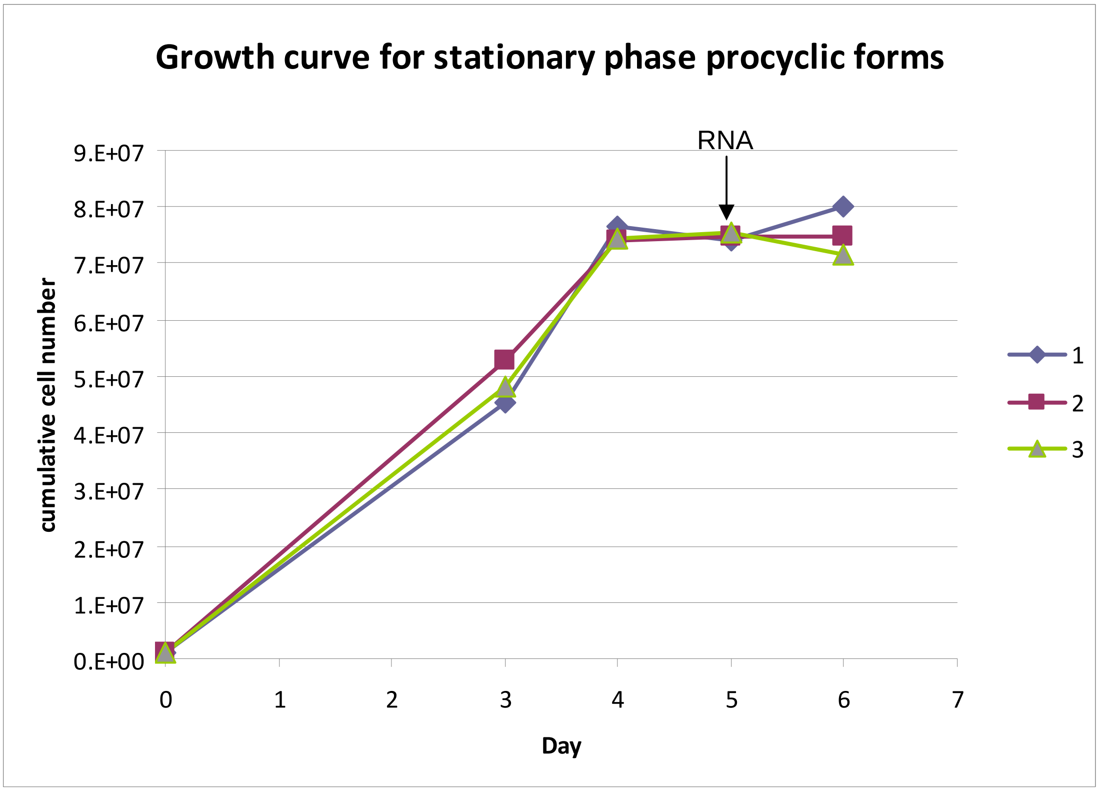

Supplement: Additional file 1 — Growth curve for PF. Growth curve showing cell densities at time of harvest of procyclic forms. [file 1471-2164-10-482-S1.PDF]
