# Supplementary material for: Widespread variation in transcript abundance within and across developmental stages of Trypanosoma brucei
Source: BMC Genomics. 2009 Oct 19;10:482. doi: 10.1186/1471-2164-10-482 (PMC2771046; doi:10.1186/1471-2164-10-482)
Supplement: Additional file 5 — Slender BF vs cBF. Genes differentially expressed in cBF versus slender BF [file 1471-2164-10-482-S5.PDF]

Additional file 5. Jensen et al. Genes showing expression changes between dividing bloodstream forms cultured in vitro or isolated from rats

Increased in slender bloodforms from animals

SysID	Description	Fold change	q-value	Slender BF signal
Tb927.5.1390	64 kDa invariant surface glycoprotein	1.6	0.00	11988
Tb927.5.1410	64 kDa invariant surface glycoprotein	1.5	12.8	8554
Tb927.5.1430	64 kDa invariant surface glycoprotein	1.8	0.00	11573
Tb927.7.3180	mu-adaptin 1 adaptor complex medium subunit	1.8	14.8	15073

Additionally hypothetical conserved Tb927.7.2780 (1.6-fold, q=14.8). VSG genes are not listed.

Increased in cultured bloodstream forms

SysID	Description	Fold change	q-value	cBF signal
Tb927.5.286b	adenosine transporter 1	1.6	7.9	9396
Tb10.70.3710	aspartate aminotransferase	1.7	7.9	10132
Tb927.7.4070	calpain-like cysteine peptidase	1.6	14.8	9952
Tb11.18.0006	carnitine O-acetyltransferase	1.7	10.8	8028
Tb927.3.1700	diacylglycerol acyltransferase	1.5	7.9	13625
13J3.10	ESAG6 transferrin receptor subunit	5.9	10.8	2409
Tb11.47.0012	glutaredoxin	1.6	12.8	8260
Tb10.70.5820	hexokinase, HK1	1.5	7.9	27920
Tb927.4.4520	hypothetical conserved, cold shock DNA binding domain	1.8	7.9	11127
Tb927.8.7820	hypothetical conserved, cold shock DNA binding domain	1.6	7.9	30733
Tb09.211.1720	hypothetical conserved, zinc finger	1.7	7.9	18066
Tb10.70.2020	hypothetical conserved, zinc finger	1.6	14.8	8391
Tb927.7.6580	hypothetical protein, aposialoprotein motif	1.6	7.9	11080
Tb927.7.5920	mercaptopyruvate sulfurtransferase	1.5	14.8	3419
Tb927.7.5820	monooxygenase	1.6	10.8	5284
Tb10.70.3720	NADH-dependent fumarate reductase	1.5	9.9	8084
Tb11.52.0003	oligopeptidase b	1.5	7.9	13945
Tb927.4.3770	protein kinase , CAMK group	1.7	7.9	11977
Tb927.6.3890	replication factor C subunit 2	1.6	7.9	11375
Tb927.1.4780	serine peptidase	1.9	14.8	2732
Tb09.211.1020	sphingomyelin synthase family SLS2	1.7	7.9	9771
Tb10.61.2520	TFIIF-stimulated CTD phosphatase	1.7	10.8	16649
Tb927.3.5090	tryparedoxin	1.9	7.9	14585
Tb927.3.1500	VSG-related VR5 cluster 2.2	1.9	9.9	13722

Additionally hypothetical and conserved hypothetical proteins Tb927.3.2460, Tb927.3.5200, Tb927.4.2790, Tb927.6.2560, Tb09.211.1620, Tb927.7.1770, Tb927.7.2180, Tb927.7.4050, Tb927.7.5830, Tb927.7.5850, Tb927.7.6490, Tb927.7.6570, Tb10.70.2840, Tb10.70.4080, Tb10.70.5450, Tb10.6k15.0600, Tb10.6k15.2690, Tb11.01.4040, Tb11.47.0025 were all 1.5-1.9 fold up regulated in cBF with a q value <15. VSG genes are not listed.
